# Talcoma: A Diagnostic Challenge in Differential Diagnosis of Pleural Masses

**DOI:** 10.1155/2015/652760

**Published:** 2015-07-26

**Authors:** Iclal Ocak, Rohit Dewan

**Affiliations:** University of Pittsburgh Medical School Presbyterian, Radiology Suite 200 East Wing, 200 Lothrop Street, Pittsburgh, PA 15213, USA

## Abstract

Talcoma is a pleural mass which may develop as a rare complication following talc pleurodesis. Talc pleurodesis is performed to obliterate the pleural space to prevent recurrent pleural effusions or persistent pneumothoraces. The present report describes a case of a patient who developed enlarging pleural mass (talcoma) following talc pleurodesis.

## 1. Introduction

Talcoma is a pleural mass which may rarely develop as a complication to talc pleurodesis. Talc pleurodesis is a procedure performed to obliterate the pleural space to prevent recurrent pleural effusions or persistent pneumothoraces. Pleurodesis is commonly performed by draining the pleural fluid, followed by either a mechanical procedure (abrasion, or partial pleurectomy) or instillation of a chemical irritant into the pleural space. The resulting inflammation and fibrosis prevent further accumulation of air and fluid within the potential pleural space. Talc is the most effective sclerosant available for chemical pleurodesis involving malignant pleural effusions [1]. When compared to indwelling pleural catheter placement, talc pleurodesis has been shown to be equally effective in relieving dyspnea [1].

The present report describes a case of a patient who developed a slowly enlarging pleural mass (talcoma) over the course of 16 years following talc pleurodesis for recurrent pneumothoraces.

## 2. Case Report

A 28-year-old male with history of immunodeficiency secondary to Hyperimmunoglobulin E syndrome (Job syndrome) had a complex surgical history including bilateral video assisted thoracic surgery (VATS) with bullae resections secondary to multiple pulmonary infections and recurrent pneumothoraces at an outside hospital. The left hemithorax bulla resection was complicated by lack of reexpansion requiring talc pleurodesis.

**Figure 1:**
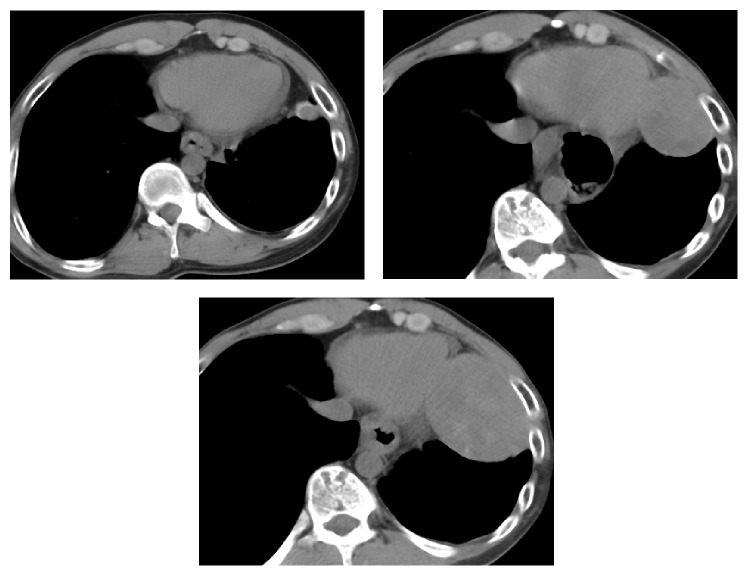
Noncontrast chest CT shows enlarging heterogeneous, well defined mass lesion at the level of left cardiophrenic recess (consecutive CTs from 2006, 2011, and 2013). The patient also had a hiatal hernia characterized by dilation and wall thickening of the distal esophagus. This was treated with Nissen fundoplication in 2012.

**Figure 2:**
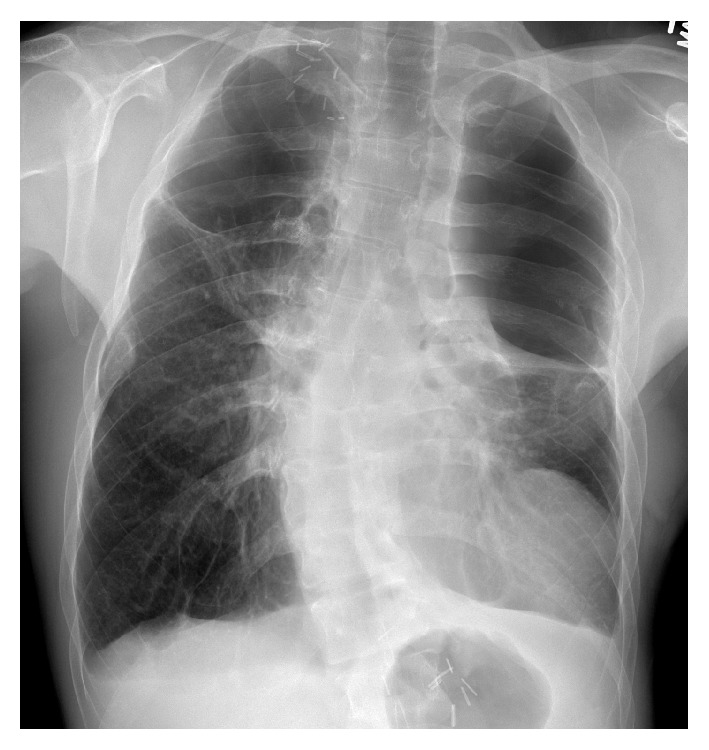
PA chest X-ray taken in July 2013 shows mass lesion at the level of left cardiophrenic recess and biapical large bullae.

He presented to us approximately 8 years after talc pleurodesis with a chest CT to be evaluated for pneumonia. His chest CT demonstrated postoperative changes from prior bullectomy with recurrent large bullae occupying the bilateral lung apices. A well-circumscribed heterogeneous soft tissue density mass was noted at the left cardiophrenic angle measuring 2.4 × 1.8 cm (Figure 1). As there were no definite malignant features, this mass was followed as the patient returned for multiple chest CTs for recurrent infections. Approximately 3 years later in 2009 the mass had increased in size measuring 3.7 × 2.7 cm. There was a further increase in size in 2011 where the mass had grown to 5.5 × 4.4 cm (Figure 1). In 2013, the mass had grown to 7.5 × 6 cm and there were large apical bullae (Figures 1 and 2).

**Figure 3:**
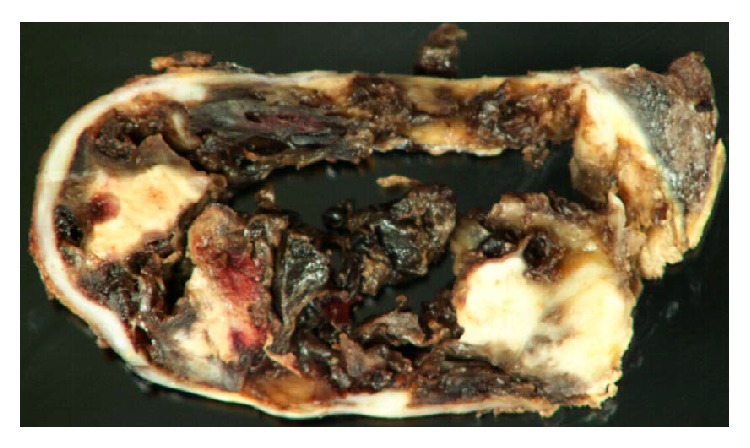
Gross pathology images show cystic hemorrhagic mass, due to talcoma.

Surgical resection was advocated as the mass demonstrated considerable growth over the years. A left-sided thoracotomy was performed for excision of this pleural mass, during which significant pleural adhesions were noted from prior pleurodesis. The mass was dissected free from the adherent pericardium. Pathology demonstrated chronic pleuritis, calcifications, and a foreign body giant cell reaction secondary to talc pleurodesis (Figure 3).

## 3. Discussion

Talc pleurodesis is a widely used procedure in the treatment of recurrent pneumothoraces and pleural effusions with a low recurrence rate. Talc pleurodesis has also been shown to be effective in treating recurrent benign and malignant pleural effusions, thus allowing the rare diagnosis of talcoma to be seen in multiple patient populations.

Ahmed and Shrager reported a young woman with a large, calcified anterior mediastinal mass discovered 18 months following a left talc pleurodesis. The lesion was evaluated and treated as the thymoma or teratoma with excision by a transcervical approach. Pathologic examination revealed a giant talc granuloma [2]. In our case the pleural mass developed in 2006 approximately 8 years after talc pleurodesis, at which time it measured approximately 2 cm. In 2013, approximately 15 years after talc pleurodesis, the mass had grown to 8 cm.

When talc particles are infused into the pleural space, they are unable to enter the alveoli or systemic circulation [3]. These particles commonly deposit along the posterior basal pleural space but can be seen paramediastinally along the pericardium extending up to the apex or within the fissures [3]. Radiographic changes following the procedure include obliteration of the costophrenic angle, or occasionally pleural thickening with calcification [4]. Chest CT demonstrates calcified pleural thickening after talc pleurodesis with focal areas of high attenuation measuring up to 320 Hounsfield units [3]. PET-CT demonstrates high FDG uptake and hypermetabolism secondary to the induced granulomatous reaction and can last for more than 20 years [4]. History is important, as these nonspecific imaging findings can also be seen with asbestos exposure, early stage malignant pleural mesothelioma, and metastatic disease.

## 4. Conclusion

The diagnostic challenge of evaluating a talcoma arises from its nonspecific imaging characteristics. Calcified pleural thickening and obliteration of the costophrenic angle with increased FDG avidity on PET-CT may be the only diagnostic clues. Although it has only been rarely observed after talc pleurodesis, the possibility of talcoma should still be considered in the differential diagnosis of pleural masses with patients that have a remote history of talc pleurodesis.

## References

[1] Davies H. E., Mishra E. K., Kahan B. C. (2012). Effect of an indwelling pleural catheter vs chest tube and talc pleurodesis for relieving dyspnea in patients with malignant pleural effusion: the TIME2 randomized controlled trial. *Journal of the American Medical Association*.

[2] Ahmed Z., Shrager J. B. (2003). Mediastinal talcoma masquerading as thymoma. *Annals of Thoracic Surgery*.

[3] Williams T., Gostelow B., Woods D., Spyt T. (1998). Apical pleural mass developing following talc pleurodesis. *Respiratory Medicine*.

[4] Vandemoortele T., Laroumagne S., Roca E. (2014). Positive FDG-PET/CT of the pleura twenty years after talc pleurodesis: three cases of benign talcoma. *Respiration*.

